# Body Attunement: An Integrative Framework for Embodied Awareness and Therapeutic Presence in Counseling

**DOI:** 10.3390/bs16091523

**Published:** 2026-08-29

**Authors:** Rebecca Taylor, Sara Wood, Eleanor Jackson

**Affiliations:** School of Counseling, College of Adult and Graduate Studies, Colorado Christian University, Lakewood, CO 80226, USA

**Keywords:** body attunement, body image, embodiment, counselor education, eating disorders

## Abstract

Body image scholarship has substantially advanced understanding of how individuals evaluate, relate to, and experience their bodies, particularly within eating disorder research. However, existing constructs such as negative body image, positive body image, body neutrality, embodiment, interoception, and somatic awareness do not fully explain the clinical process through which bodily information is noticed, interpreted, integrated, and intentionally used within counseling. This conceptual article synthesizes the literature from body image research, embodiment theory, interoception research trauma-informed and somatic psychotherapy, and counselor education to propose Body Attunement as a potentially distinct psychological construct for counselor education. Body Attunement is provisionally defined as the counselor’s capacity to perceive, reflect upon, integrate, and intentionally respond to their own embodied information during the therapeutic encounter in conjunction with other relevant clinical information. Potential relationships with therapeutic presence, emotional regulation, relational attunement, and client outcomes are proposed as areas for future empirical study. A preliminary clinical process model is presented, including noticing, curiosity, integration, intentional response, and reflection. The construct is discussed as a potential dimension of embodied clinical competence with particular relevance for counselor education and eating disorder treatment. Future qualitative and quantitative research is needed to refine the construct, develop assessment tools, and examine its relationship to therapeutic presence, emotional regulation, counselor self-awareness, and client outcomes.

## 1. Body Attunement: An Integrative Framework for Embodied Awareness and Therapeutic Presence in Counseling

Perhaps one of the greatest paradoxes of being human is that we spend our entire lives living within a body while often remaining disconnected from it. We learn to evaluate it, compare it, discipline it, improve it, and sometimes even criticize it, yet we receive remarkably little guidance on how to understand it. As psychology has expanded its understanding of body image, another question quietly emerges: What if the body is more than something to evaluate? What if it is also something to which we can learn to listen? Most of the client’s I work with have learned to actively avoid listening to their body. As one client told me: “In my head, my body image issues go to the same place where the HOA letters go!” (Meaning… straight in the trash).

Body image has become one of the most extensively studied constructs within psychology, particularly within the eating disorder literature (Cash, 2004; Cash & Smolak, 2011; Tylka & Wood-Barcalow, 2015a). Over the past four decades, these researchers have substantially advanced our understanding of how individuals think, feel, perceive, and behave in relation to their bodies. Body image is now widely recognized as a multidimensional construct encompassing cognitive, affective, perceptual, and behavioral components rather than simply one’s satisfaction with physical appearance (Cash, 2004; Cash & Smolak, 2011).

Recent scholarship has fundamentally shifted the field of body image literature by demonstrating that positive body image is not merely the absence of negative body image (Tylka & Wood-Barcalow, 2015a, 2015b). Rather, positive body image represents a distinct psychological construct characterized by body appreciation, body acceptance, respect for bodily needs, adaptive self-care, and gratitude toward the body. The distinction in the literature that negative body image and positive body image are separate constructs has redirected the field from an exclusive focus on reducing pathology toward understanding the psychological processes that promote flourishing (Tylka & Wood-Barcalow, 2015a).

While the body image literature has significantly advanced our clinical understanding of a client’s body image thoughts, the research reveals an important conceptual gap. Much of the existing body image literature focuses on how individuals evaluate their bodies (think about their bodies). More recent literature has given attention to how individuals relate to their bodies in the present moment and how that relationship influences emotional regulation, therapeutic presence, and relational functioning. So how do counselors attend to and integrate their own embodied information during the therapeutic encounter? This paper serves to do just that.

Parallel developments within trauma treatment, embodiment research, mindfulness, interoception, and affective neuroscience have increasingly emphasized the role of bodily processes in psychological functioning. Peter Levine’s work on trauma (Levine, 1997, 2010), Stephen Porges’s controversial Polyvagal Theory (Porges, 2011), Daniel Siegel’s Interpersonal Neurobiology (Siegel, 2012), and the growing literature on interoception collectively suggest that the body is not merely an object of evaluation but an ongoing source of psychological information. Across these perspectives, bodily experience is understood as integral to emotional regulation, self-understanding, and interpersonal connection.

Despite these converging bodies of literature, no existing construct fully integrates body image research with embodied clinical awareness in a manner directly applicable to counselor education and psychotherapy. Existing concepts (including embodiment, interoception, mindfulness, and somatic awareness) each explain important aspects of bodily experience (Craig, 2002; Fuchs & Koch, 2014; Shapiro et al., 2006) but do not fully capture the counselor’s and client’s ongoing relationship with their own body during the therapeutic encounter.

This paper proposes Body Attunement as a potentially distinct counselor-focused construct describing the capacity to intentionally notice, reflect upon, integrate, and respond to one’s own embodied information during the therapeutic encounter while considering that information alongside other relevant clinical information. Rather than replacing existing constructs, Body Attunement is proposed as an integrative framework that builds upon prior research (specifically interoception) while offering a counselor-specific lens through which embodied clinical competence may be conceptualized, cultivated, and empirically examined. Simply put, this paper proposes that counselors’ own embodied experiences may represent one source of clinical information that requires reflective and ethical consideration.

## 2. Beyond Positive and Negative Body Image

Historically, body image research focused predominantly on pathology. Early scholarship emphasized body dissatisfaction, appearance anxiety, distorted body perception, and eating disorder symptomatology, consistently demonstrating that negative body image is associated with depression, anxiety, disordered eating, lower self-esteem, impaired psychosocial functioning, and increased risk for eating pathology (Cash & Pruzinsky, 2002; Cash & Smolak, 2011; Stice & Shaw, 2002; Grogan, 2022). While this work substantially advanced understanding of body image disturbance, it largely conceptualized health as the reduction or absence of pathology.

Over the past two decades, researchers have challenged this deficit-oriented perspective by demonstrating that positive body image is not simply the opposite end of a body image continuum (Tylka & Wood-Barcalow, 2015a). Rather, positive body image is a distinct, multidimensional psychological construct characterized by body appreciation, acceptance, respect, adaptive appearance investment, body functionality appreciation, protective filtering of sociocultural messages, and engagement in health-promoting self-care behaviors (Avalos et al., 2005; Tylka & Wood-Barcalow, 2015a, 2015b; Webb et al., 2015). This conceptual shift represented a significant advancement in the field, redirecting attention from reducing body dissatisfaction to understanding the characteristics that promote psychological flourishing and resilience.

Qualitative investigations of individuals who report positive body image further illustrate that these individuals do not necessarily experience perfect satisfaction with their appearance (Wood-Barcalow et al., 2010). Rather, they describe a flexible and compassionate relationship with their bodies characterized by acceptance, appreciation of body functionality, realistic expectations, and the ability to navigate inevitable fluctuations in body image without becoming consumed by them (Frisén & Holmqvist, 2010; Wood-Barcalow et al., 2010). Positive body image, therefore, reflects an adaptive relationship with one’s body rather than perpetual positive feelings toward it.

More recently, body neutrality has emerged in popular culture and clinical conversations as an alternative approach to body image. Body neutrality encourages individuals to reduce the emphasis placed on appearance and instead focus on respecting the body, appreciating its functionality, and recognizing that self-worth extends beyond physical appearance (Alleva et al., 2015; Tylka & Wood-Barcalow, 2015a; Wood-Barcalow et al., 2024). A central theme of body neutrality is moving towards appreciating what the body does for you, rather than how it looks (Alleva et al., 2015; Wood-Barcalow et al., 2024). Although body neutrality has gained considerable attention, leading body image scholars argue that it is not a novel construct but rather reflects principles that have long been embedded within positive body image research, including body appreciation, functionality appreciation, body image flexibility, self-care, and body respect (Wood-Barcalow et al., 2024). Rather than replacing positive body image, body neutrality may best be understood as one practical orientation through which individuals cultivate many of the same adaptive characteristics described within the positive body image literature.

The growing interest in body neutrality nevertheless highlights an important clinical observation: individuals often benefit from reducing evaluative judgments about their bodies, whether those judgments are highly negative or characterized by pressure to consistently “love” their bodies. A less evaluative stance may create greater psychological flexibility and reduce the cognitive preoccupation that interferes with emotional awareness and self-regulation. This concept is particularly relevant within counseling, where clinicians are trained to recognize and bracket personal biases in order to remain open, curious, and responsive to clients’ experiences. Yet counselor education has devoted remarkably little attention to helping counselors develop a similarly nonjudgmental relationship with their own bodily experiences.

The positive body image and body neutrality literature has substantially advanced our understanding of how individuals relate to their bodies (Tylka & Wood-Barcalow, 2015a; Webb et al., 2015; Wood-Barcalow et al., 2024). However, these constructs primarily describe the quality of one’s relationship with the body: whether that relationship is characterized by appreciation, acceptance, flexibility, or respect. Less attention has been given to the dynamic process through which individuals notice, interpret, and respond to bodily information in real time. Appreciating the body is not necessarily synonymous with listening to it. For example, most would agree that a parent can appreciate their child, while at the same time, not listen to them. This conceptual distinction may be particularly important for counselors, for whom the relationship between bodily awareness, emotional regulation, therapeutic presence, and relational attunement warrants further examination.

## 3. From Body Evaluation to Embodied Experience

The emergence of positive body image shifted the field away from pathology and toward psychological flourishing. Yet, even this important advancement continues to emphasize how individuals evaluate or relate to their bodies. Positive body image focuses on appreciation, acceptance, body functionality, and flexible responses to sociocultural appearance pressures (Tylka & Wood-Barcalow, 2015a, 2015b). These constructs represent a healthier relationship with the body, but they remain largely concerned with one’s beliefs, attitudes, and evaluations of bodily experience.

The embodiment literature represents a further conceptual shift by moving beyond body evaluation altogether. Rather than viewing the body as an object to be judged, embodiment conceptualizes human experience as fundamentally grounded in bodily processes. Thoughts, emotions, memory, perception, identity, and interpersonal relationships emerge through the continuous interaction of the brain, body, and environment (Fuchs & Koch, 2014). Within this perspective, the body is not simply something individuals possess but the primary medium through which they experience themselves and engage with the world.

Within eating disorder research, Piran’s Developmental Theory of Embodiment significantly advanced this perspective by proposing that healthy embodiment develops through experiences of agency, body connection, attuned self-care, and resistance to objectification (Piran, 2016, 2017). Rather than emphasizing appearance satisfaction alone, positive embodiment involves inhabiting one’s body as a trusted source of experience and action. Similarly, Cook-Cottone’s Embodied Self Model conceptualizes psychological well-being as emerging through attuned self-care, mindful awareness, and integration of physical, emotional, cognitive, relational, and spiritual experiences (Cook-Cottone, 2015, 2020). Collectively, these models emphasize that health involves living through the body rather than evaluating it from the outside.

Likewise, a growing body of research suggests that how individuals inhabit and engage with their bodies may be just as important as how they evaluate them. Embodiment has been associated with healthier psychological functioning and adaptive relationships with the body (Halliwell, 2015; Piran, 2017; Webb et al., 2015), while empirical studies have demonstrated that embodied processes may serve as important mechanisms linking relational experiences and eating disorder psychopathology (Monteleone et al., 2017) and that adaptive body image processes contribute to psychological well-being through healthier coping strategies (Matera et al., 2024). Collectively, these findings suggest that psychological health may be influenced not only by what individuals think about their bodies but also by how they experience, inhabit, and respond to bodily experience.

Although embodiment has become an influential framework, it remains an exceptionally broad construct. Embodiment encompasses movement, identity, culture, sexuality, social interaction, agency, body awareness, environmental engagement, and lived experience (Fuchs & Koch, 2014; Piran, 2017). This breadth is one of embodiment’s greatest strengths because it provides a comprehensive understanding of human functioning. At the same time, its scope presents a challenge for counselor education. Embodiment offers a valuable theoretical foundation but provides relatively little specificity regarding how counselors intentionally develop awareness of their own bodily experiences during clinical work or how they utilize those experiences to enhance therapeutic presence and decision-making. The proposed Body Attunement framework seeks to conceptualize how counselors identify and reflect upon their own embodied experiences during therapeutic encounters and how such information might be integrated ethically into clinical decision-making. Furthermore, the paper is proposing body attunement as a distinct construct that describes the intentional capacity to notice, interpret, and respond to embodied information within the context of clinical practice.

Counselor educators increasingly recognize the importance of preparing counselors who are emotionally present, relationally attuned, and congruent with clients. Rogers (1961) described this quality as congruence: the therapist’s capacity to remain genuine, aware, and authentically present within the therapeutic relationship. Likewise, the contemporary embodiment literature emphasizes that effective therapeutic presence is grounded not only in cognitive understanding but also in lived bodily awareness (Geller & Greenberg, 2012). Therapeutic presence is referred to as a counselor’s ability to remain fully engaged, grounded, and emotionally available (Geller & Greenberg, 2012). Geller and Greenberg (2012) also mention that therapeutic presence is essential for effective treatment in counseling. The argument could be made that to help clinicians in their training and development, educators are called to ensure therapists are equipped to know how to engage and build therapeutic presence. However, based on our definition of body attunement, it may not be fully possible for a clinician to do so if they do not have strong body attunement because they might not even be able to be fully engaged in the cues their body is sending them (interoception), or fully present (mindfulness), or emotionally available, or able to translate their emotional signals from their body (embodiment).

So the pedagogical question remains: how does one teach embodiment? Because embodiment is a broad and multifaceted construct, it may be difficult to operationalize within counselor education. The present paper proposes that Body Attunement offers one teachable pathway toward embodied clinical practice by providing counselors with concrete skills for intentionally noticing, interpreting, and responding to their own bodily experiences during therapeutic encounters.

As counselors become increasingly attuned to their own embodied experience, they may also become better equipped to help clients develop a similar relationship with their own bodies. This process parallels the longstanding counseling skill of immediacy. Traditionally, immediacy refers to the counselor’s intentional use of what is occurring within the therapeutic relationship in the present moment to deepen awareness, strengthen the therapeutic alliance, and facilitate growth (Egan & Reese, 2019; Ivey et al., 2018). Body Attunement extends this principle inward by encouraging counselors to attend not only to what is happening between counselor and client but also to what is occurring within the counselor’s own embodied experience. Physiological sensations, shifts in posture, muscle tension, breathing patterns, or emotional activation become valuable sources of clinical information that, when thoughtfully interpreted rather than reactively acted upon, may enhance therapeutic presence, clinical judgment, and relational attunement (Halonen et al., 2025). In this way, the counselor’s body becomes another source of information within the therapeutic encounter, complementing traditional skills of observation, reflection, and immediacy.

## 4. Somatic Awareness, Interoception, and the Listening Body

Trauma theory further extends embodiment by emphasizing that psychological experience is fundamentally physiological. Levine (1997, 2010) argues that trauma is not simply stored as cognitive memory but is expressed through dysregulated nervous system responses that persist within the body. Consequently, healing requires more than cognitive insight; it requires individuals to gradually reconnect with bodily sensations that have become fragmented, avoided, or chronically dysregulated. Similarly, van der Kolk (2014) proposes that “the body keeps the score,” illustrating how traumatic experiences are encoded within physiological systems and emphasizing that recovery depends upon restoring awareness of internal bodily experience.

These perspectives have contributed substantially to the growing emphasis on somatic awareness within psychotherapy. Somatic awareness generally refers to the capacity to notice internal bodily sensations such as breathing, muscle tension, posture, movement, temperature, visceral sensations, and autonomic activation. Such awareness provides valuable information regarding emotional states, stress responses, and nervous system regulation (McConnell, 2020; Payne et al., 2015; Siegel, 2012). Within Somatic Internal Family Systems (IFSs), McConnell (2020) further emphasizes that cultivating curiosity toward bodily sensations allows individuals to differentiate internal experiences, recognize protective responses, and access greater self-energy, illustrating how somatic awareness can become an active component of therapeutic presence rather than merely a passive observation of bodily states. Closely related is the construct of interoception, which refers to the perception of internal physiological signals originating from within the body (Khalsa et al., 2018). Contemporary neuroscience identifies interoception as fundamental to emotional awareness, self-regulation, and decision-making because signals related to hunger, satiety, heart rate, respiration, pain, fatigue, and visceral sensations continuously inform the brain about the body’s internal condition (Craig, 2002, 2009). Siegel (2012) similarly describes interoception as one of the mind’s primary mechanisms for developing self-awareness, emotional integration, and interpersonal attunement.

Interoceptive awareness has also been conceptualized as multidimensional. Mehling et al. (2012) developed the Multidimensional Assessment of Interoceptive Awareness (MAIA) to assess several dimensions of an individual’s relationship with internal bodily experience, including noticing bodily sensations, attention regulation, emotional awareness, self-regulation, body listening, and trusting bodily signals. These dimensions demonstrate that interoception extends beyond the simple detection of physiological sensations and includes how individuals attend to and respond to internal bodily information. Garfinkel et al. (2015) further distinguish interoceptive accuracy, interoceptive sensibility, and interoceptive awareness. These frameworks substantially overlap with several processes included in the proposed Body Attunement construct, particularly noticing, curiosity toward bodily experience, and aspects of self-regulation. Therefore, Body Attunement is not proposed as an alternative to, or replacement for, interoceptive awareness. Rather, interoceptive awareness may represent an important foundation upon which the proposed Body Attunement process builds. Its potential contribution lies in the counselor-specific clinical integration and intentional use of the counselor’s own embodied information within the therapeutic encounter. Specifically, Body Attunement is proposed as a counselor education framework for helping clinicians consider bodily information alongside cognitive, emotional, relational, contextual, and ethical information before determining whether and how that information should inform a clinical response.

Predictive-processing accounts further complicate a simple bottom-up understanding of interoception. Rather than conceptualizing bodily experience as a direct readout of physiological signals, interoceptive inference models propose that bodily experience emerges through an ongoing interaction between ascending physiological information and predictions or expectations about the body (Barrett & Simmons, 2015; Seth & Friston, 2016). Within these models, discrepancies between predicted and incoming bodily information generate prediction errors that may contribute to updating internal models of bodily experience. This perspective is particularly relevant to the proposed Body Attunement framework because a counselor’s bodily response cannot be assumed to provide direct or inherently accurate information about a client or therapeutic interaction. Bodily experiences may reflect the counselor’s physiology, previous experiences, expectations, emotional responses, relational context, or some combination of these factors. Accordingly, the Integration dimension of Body Attunement is not intended to represent the decoding of a bodily signal as fact, but rather the reflective consideration of embodied information alongside alternative explanations and other sources of clinical information before determining whether and how it should inform a response.

Mindfulness also overlaps with several elements of the proposed Body Attunement framework. Mindfulness commonly involves intentional attention to present-moment experience with an open and nonjudgmental stance, making the proposed dimensions of Notice and Curiosity particularly consistent with established mindfulness processes (Shapiro et al., 2006). Accordingly, these dimensions are not proposed as novel psychological operations unique to Body Attunement. Rather, mindful awareness may represent another foundational process upon which Body Attunement builds. The proposed contribution of Body Attunement lies in extending mindful attention to bodily experience into a counselor-specific process of clinical integration and intentional response. Within this framework, noticing bodily experience with openness is only an initial step; the counselor subsequently considers that information alongside cognitive, emotional, relational, contextual, and ethical information before determining whether and how it should influence clinical action.

Existing theories of embodiment, interoception, and somatic awareness suggest that meaningful psychological information is carried through the body (Levine, 2010; McConnell, 2020; Piran, 2017; Siegel, 2012). However, these frameworks primarily describe aspects of bodily experience rather than the clinical process through which bodily information is recognized, interpreted, integrated, and intentionally used within psychotherapy. While these constructs provide an essential theoretical foundation, they stop short of articulating how counselors can transform embodied experiences into clinically meaningful interventions.

One notable exception is the work of McConnell (2020), whose Somatic Internal Family Systems (IFS) model emphasizes cultivating awareness of bodily sensations with curiosity, compassion, and Self-energy. Rather than viewing bodily sensations as symptoms to eliminate, McConnell encourages therapists and clients to notice internal bodily experiences, distinguish among somatic cues, and explore the meaning these sensations may hold within the therapeutic process. Her work offers a clinical illustration of how bodily awareness may be explored as a potential source of psychological insight and therapeutic change. Importantly, Somatic IFS remains an emerging clinical approach, and empirical research examining its mechanisms and effectiveness is currently limited. Accordingly, it is included here as a conceptual and clinical illustration rather than as an empirically established foundation for Body Attunement.

Although McConnell’s (2020) model was developed within the theoretical framework of Internal Family Systems therapy, the clinical progression it proposes (noticing bodily sensations to curiosity, integration, and intentional response) offers one conceptual parallel to the process this paper is proposing. Body Attunement is being proposed as a counselor education construct that conceptualizes this broader process of transforming embodied awareness into intentional clinical understanding and therapeutic action.

## 5. Defining Body Attunement

Existing literature has substantially advanced the understanding of body image, embodiment, interoception, and somatic awareness. However, these constructs do not fully explain the process through which individuals perceive, interpret, and intentionally respond to bodily information within themselves and in relationship with others. We propose Body Attunement as a psychological construct that extends these existing theories by emphasizing not only awareness of bodily experience but also the capacity to accurately interpret, integrate, and respond to bodily information in ways that promote psychological health and relational connection. The authors define Body Attunement as the ongoing capacity to perceive, interpret, integrate, and intentionally respond to internal bodily experiences in ways that foster self-regulation, adaptive decision-making, and authentic relational engagement. The definition and dimensions represent a preliminary conceptual synthesis derived from the literature reviewed above. They are proposed by the authors as testable propositions rather than empirically validated characteristics of an established construct. Rather than viewing bodily sensations as isolated physiological events, Body Attunement conceptualizes the body as an active source of psychological, emotional, interpersonal, and clinical information.

This construct assumes that bodily awareness alone is insufficient. Individuals may accurately notice hunger, anxiety, muscle tension, or changes in breathing without understanding the meaning of those experiences or responding to them in adaptive ways. Body Attunement therefore represents an active process that moves beyond awareness toward interpretation, integration, and intentional action.

Within counseling, Body Attunement also extends beyond awareness of one’s own internal experience. Counselors continuously receive information through subtle bodily responses that emerge within therapeutic relationships, including changes in posture, breathing, muscular tension, emotional resonance, and physiological activation. When these experiences are interpreted thoughtfully rather than reacted to automatically, they become clinically meaningful sources of information that may enhance empathy, case conceptualization, therapeutic presence, and intervention planning.

Body Attunement is proposed here as an intrapersonal, counselor-focused process. Its primary focus is the counselor’s awareness and intentional use of their own embodied information during the therapeutic encounter. Although counselors’ embodied responses necessarily occur within relational contexts, Body Attunement does not refer to accurately reading the client’s bodily state or to cultivating the client’s interoceptive awareness. Rather, potential effects on relational attunement, therapeutic presence, client body awareness, or treatment processes are considered downstream implications of the proposed construct and require empirical examination.

Rather than replacing existing theories of embodiment or somatic awareness, Body Attunement is intended to build upon them by describing the dynamic process through which bodily information becomes psychologically meaningful and therapeutically useful.

The clinical dimensions presented in Table 1 are intended as a preliminary conceptual framework rather than a validated model. Although presented sequentially for clarity, Body Attunement is conceptualized as an iterative process in which counselors move fluidly among noticing, curiosity, integration, intentional responding, and reflective learning. These dimensions draw conceptually from research on embodiment and interoception, somatic psychotherapy, and clinical approaches that emphasize reflective curiosity toward bodily experience, including McConnell’s Somatic IFS framework. These clinical parallels are offered as conceptual influences rather than evidence validating the proposed dimensions. Future empirical work should examine whether these dimensions accurately represent counselors’ lived experiences and whether additional dimensions emerge through qualitative investigation.

## 6. Clinical and Counselor Education Implications

If future research supports Body Attunement as a meaningful construct, it may also have value as a dimension of clinical competency that could be intentionally cultivated throughout counselor education and professional development. Counselor preparation programs have long emphasized the importance of counselor self-awareness, reflective practice, multicultural humility, ethical decision-making, and interpersonal competence as foundational characteristics of effective clinical practice (CACREP, 2024; American Counseling Association [ACA], 2014). Students are encouraged to examine their personal values, biases, cultural identities, emotional reactions, and relational patterns in order to reduce their potential influence on the therapeutic relationship. Although these competencies are well established within counselor education, comparatively little attention has been devoted to intentionally developing counselors’ awareness of their own embodied experiences and the ways physiological information may influence clinical judgment, therapeutic presence, and relational attunement.

The importance of counselor self-awareness has been consistently supported throughout psychotherapy research (CACREP, 2024; Norcross & Lambert, 2019; Rogers, 1961; Skovholt & Rønnestad, 2003). Across theoretical orientations, therapist characteristics, including empathy, emotional regulation, presence, and the quality of the therapeutic alliance, remain among the strongest predictors of successful treatment outcomes (Norcross & Lambert, 2019; Wampold & Imel, 2015). Similarly, interpersonal neurobiology suggests that counselors continuously communicate emotional states through nonverbal and physiological processes that influence relational safety and co-regulation (Siegel, 2012; Siegel & Drulis, 2023). These findings suggest that effective counseling extends beyond the application of interventions to include the counselor’s moment-to-moment capacity to remain emotionally regulated, interpersonally present, and responsive within the therapeutic relationship. Body Attunement extends this perspective by proposing that embodied awareness is not simply a wellness practice but a professional competency that may strengthen these common therapeutic factors.

Current counselor education curricula understandably devote substantial attention to cognitive conceptualization, case formulation, diagnostic reasoning, ethical standards, theoretical orientation, and microskill training. While these competencies remain essential, they may insufficiently prepare counselors to recognize how their own physiological experiences contribute to clinical decision-making. Throughout psychotherapy, counselors continuously experience subtle bodily cues (including changes in breathing, muscle tension, posture, heart rate, visceral sensations, and patterns of activation) that may communicate important information regarding their own emotional regulation, interpersonal resonance, emerging countertransference, or the client’s emotional state. Body Attunement proposes that these physiological experiences are not distractions from therapy but potentially meaningful clinical information when approached with curiosity, reflection, and intentionality.

Within this framework, Body Attunement may be understood as one dimension of embodied clinical competence: the counselor’s capacity to intentionally recognize, interpret, integrate, and respond to embodied information in ways that enhance therapeutic presence, emotional regulation, ethical decision-making, and relational attunement. Whereas traditional conceptions of clinical competence emphasize theoretical knowledge, diagnostic reasoning, multicultural responsiveness, ethical practice, and interpersonal microskills, embodied clinical competence acknowledges that effective counseling also depends upon the therapist’s ability to utilize physiological information as a meaningful source of clinical understanding. Body Attunement is proposed as one foundational process through which this broader competency develops, providing counselors with an additional avenue for understanding themselves, their clients, and the therapeutic relationship.

Developing Body Attunement may require experiential pedagogical approaches that complement traditional didactic instruction. Experiential learning has long been recognized as an essential component of counselor development because professional identity is cultivated not only through knowledge acquisition but also through reflective practice and lived clinical experience (Kolb, 1984; Stoltenberg & McNeill, 2010). Building upon this tradition, counselor educators may intentionally incorporate body-based reflective practices such as mindfulness, guided body awareness, experiential movement, somatic supervision, interpersonal process groups, reflective journaling, and structured self-observation into counselor training. These experiences are not intended simply to promote student wellness; rather, they provide opportunities for counselors-in-training to strengthen their ability to perceive, interpret, integrate, and intentionally respond to embodied information as it emerges during therapeutic encounters.

Important to note, Body Attunement is not proposed as another counseling theory or treatment model. Rather, it is conceptualized as a foundational therapist characteristic that may enhance counselors’ ability to implement a wide range of theoretical orientations with greater emotional regulation, therapeutic presence, and relational responsiveness. McConnell’s (2020) Somatic Internal Family Systems framework offers one clinical example of cultivating curiosity toward embodied experience while remaining grounded in Self-energy. Although empirical support for Somatic IFSs is still developing, Body Attunement draws conceptually from this emphasis on reflective curiosity while proposing a broader process intended to be examined across counseling theories, clinical populations, and professional training. As counselor education continues to embrace holistic, trauma-informed, and integrative models of care, Body Attunement may offer a valuable framework for preparing counselors to engage both their clients and themselves with increased presence, discernment, and embodied clinical awareness.

## 7. Implications for Eating Disorder Treatment

Eating disorder treatment provides a particularly compelling context in which to consider the clinical relevance of Body Attunement. Although body image disturbance remains a defining feature of many eating disorders, recovery requires substantially more than changing appearance-related beliefs. Clients frequently must rebuild the capacity to recognize, interpret, and respond to internal bodily experiences that have become disrupted through restriction, binge eating, purging, compulsive exercise, or chronic body surveillance. Hunger, fullness, satiety, fatigue, emotional arousal, safety, and pleasure often become unreliable or ignored sources of information, requiring intentional relearning throughout the recovery process.

Interoceptive disturbance is particularly relevant to eating-disorder research, although findings vary depending on the dimension and physiological system being examined. Systematic reviews have identified interoceptive difficulties across disordered-eating populations, while more focused research has identified alterations in gastrointestinal interoception and neural processing of interoceptive information (Martin et al., 2019; Khalsa et al., 2022). Predictive-processing accounts have also been applied to eating disorders, proposing that difficulties may arise not only in detecting bodily signals but in the relationship between expected and experienced bodily states. Importantly, interoceptive dysfunction should not be treated as a unitary deficit: recent work suggests that some dimensions, such as cardiac interoceptive accuracy in anorexia nervosa, may not differ consistently from healthy comparison groups (Gend et al., 2026). Thus, the eating-disorder literature further supports a multidimensional understanding of interoception in which noticing bodily information and determining its meaning are related but distinguishable processes.

This emphasis aligns closely with contemporary eating disorder treatment models, including intuitive eating, which encourages individuals to reconnect with internal physiological cues rather than external rules governing food and body weight (Tribole & Resch, 2020). However, helping clients reconnect with these signals requires counselors who are themselves capable of recognizing and trusting embodied information. If counselors have not developed awareness of their own physiological responses, emotional activation, and bodily experiences, they may unintentionally rely primarily on cognitive interventions while overlooking valuable somatic information emerging within the therapeutic relationship.

The value of considering Body Attunement within eating-disorder treatment is therefore not that the construct explains clients’ disrupted interoception; client interoception remains a distinct construct. Rather, eating-disorder treatment provides a particularly relevant clinical context in which to test the proposed counselor-focused Body Attunement framework. Because food, body, weight, control, hunger, fullness, and medical risk may evoke significant physiological and emotional responses within both clients and clinicians, counselors must distinguish their own embodied reactions from assumptions about the client’s internal state. Body Attunement proposes one process through which counselors might make this distinction more deliberately.

The suggested framework generates several preliminary testable hypotheses. First, among clinicians treating eating disorders, greater Body Attunement may be associated with greater ability to identify and differentiate their own embodied activation from assumptions about a client’s internal experience. Second, greater Body Attunement may be associated with more reflective recognition of counselor countertransference and greater therapeutic presence during eating-disorder treatment. Third, counselor-training interventions designed to cultivate Body Attunement may increase counselors’ ability to identify, contextualize, and deliberately respond to their own bodily experiences during simulated or actual eating-disorder encounters. These hypotheses concern counselor processes rather than presumed changes in client outcomes and require empirical testing before clinical benefit can be inferred.

## 8. Future Directions

The present article is conceptual and represents an initial effort to define Body Attunement as a distinct psychological construct. Accordingly, the proposed definition and clinical dimensions should be viewed as preliminary and subject to empirical refinement. Future research should begin with qualitative inquiry exploring how experienced counselors describe noticing, interpreting, integrating, and responding to embodied experiences during psychotherapy. Findings from these investigations may inform refinement of the construct and contribute to the development of operational definitions and measurable dimensions.

Subsequent quantitative research may then focus on developing and validating instruments capable of assessing Body Attunement among counselors-in-training and practicing clinicians. Such work could examine relationships between Body Attunement and therapeutic presence, emotional regulation, counselor self-awareness, therapeutic alliance, multicultural responsiveness, and treatment outcomes across a variety of clinical populations. Because Body Attunement is proposed as a potentially teachable clinical competency, future intervention studies could examine whether structured educational experiences, including somatic training, experiential learning, mindfulness practices, and supervision, are associated with measurable changes in counselors’ embodied clinical awareness.

## 9. Conclusions

Over the past four decades, body image scholarship has transformed our understanding of how individuals think about, evaluate, and relate to their bodies. More recently, positive body image research has demonstrated that psychological health involves far more than the absence of body dissatisfaction, while the emergence of body neutrality has highlighted the value of reducing evaluative judgments altogether. At the same time, embodiment research, interpersonal neurobiology, interoception, trauma theory, and somatic psychotherapy have collectively emphasized that the body is not merely an object of perception but a central source of emotional, physiological, and relational information.

Despite these significant advances, an important conceptual gap remains: existing constructs describe how individuals evaluate their bodies, inhabit their bodies, or become aware of bodily sensations, yet none fully capture the dynamic clinical process through which counselors intentionally perceive, interpret, integrate, and respond to embodied information within themselves and within therapeutic relationships. The proposed construct of Body Attunement seeks to address this gap by conceptualizing the body not primarily as an object of evaluation, but as a continual source of meaningful psychological information. In doing so, Body Attunement extends existing theories by emphasizing not only bodily awareness but also the intentional interpretation and clinical application of embodied experience.

Rather than replacing the well-established constructs of body image, positive body image, embodiment, interoception, mindfulness, or somatic awareness, Body Attunement is intended to complement and integrate these perspectives into a counselor-specific framework with direct relevance for psychotherapy and counselor education. As the counseling profession continues to emphasize holistic, relational, trauma-informed, and developmentally responsive care, cultivating counselors’ capacity to intentionally engage with their own embodied experiences may become an increasingly important component of clinical competence.

The present article represents an initial conceptual proposal rather than a completed theory. Future qualitative research is needed to explore how experienced counselors describe the lived experience of Body Attunement and to refine its proposed dimensions. Such work may provide the foundation for the development of valid assessment instruments and empirical investigations examining its relationship with therapeutic presence, emotional regulation, counselor self-awareness, and client outcomes. Ultimately, as psychology continues to expand beyond understanding how people evaluate their bodies toward understanding how they experience and engage with them, Body Attunement offers one possible framework for helping counselors, and the clients they serve, learn not only to appreciate the body, but to accurately listen to it.

## Figures and Tables

**Table 1:** Clinical dimension.

	Description	Example in Counseling Practice
**1. Notice**	Recognizing bodily sensations without immediate judgment or avoidance.	A counselor notices tightening in the chest while discussing a client’s trauma.
**2. Curiosity**	Remaining open to bodily experience with compassionate inquiry rather than interpretation or suppression.	Rather than ignoring the tension, the counselor wonders what the sensation may represent.
**3. Integration**	Considering bodily information alongside emotions, thoughts, relational dynamics, clinical context, and alternative explanations without assuming that the bodily response accurately represents the client’s experience.	The counselor recognizes the tension may reflect empathic resonance, countertransference, or awareness of client activation.
**4. Intentional Response**	Responding thoughtfully rather than reactively, allowing embodied information to inform therapeutic presence and intervention.	The counselor slows the pace of therapy, grounds themselves, and gently explores the client’s emotional experience.
**5. Reflection**	Reviewing embodied experiences following sessions to increase self-awareness and professional growth.	During supervision or reflective journaling, the counselor considers recurring bodily responses across clients.

## Data Availability

No new data were created or analyzed in this study. Data sharing is not applicable to this article.
